# Low HDL cholesterol and the eNOS Glu298Asp polymorphism are associated with inducible myocardial ischemia in patients with suspected stable coronary artery disease

**DOI:** 10.1186/s12872-024-03846-7

**Published:** 2024-03-22

**Authors:** Cecilia Vecoli, Chiara Caselli, Martina Modena, Giancarlo Todiere, Rosa Poddighe, Serafina Valente, Fabrizio Bandini, Andrea Natali, Lorenzo Ghiadoni, Aldo Clerico, Concetta Prontera, Simona Vittorini, Nicoletta Botto, Michele Emdin, Danilo Neglia

**Affiliations:** 1 Institute of Clinical Physiology-CNR, Via G. Moruzzi 1, Pisa, Italy; 2 Cardiovascular Department, Gabriele Monasterio Foundation, Via G. Moruzzi 1, Pisa, Italy; 3 Sant’Anna School of Advanced Studies, Pisa, Italy; 4 Ospedale Della Versilia, Lido Di Camaiore, Lucca, Italy; 5 Careggi University Hospital (AOUC), Florence, Italy; 6 Az. Sanitaria Di Firenze, Florence, Italy; 7 Department of Clinical and Experimental Medicine, University of Pisa, Pisa, Italy

**Keywords:** Endothelial dysfunction, Endothelial nitric oxide synthase, eNOS, Single nucleotide polymorphism, Obstructive coronary artery disease, Inducible myocardial ischemia

## Abstract

**Background:**

The endothelial nitric oxide synthase (eNOS) gene deficiency is known to cause impaired coronary vasodilating capability in animal models. In the general clinical population, the eNOS gene polymorphisms, able to affect eNOS activity, were associated with cardiometabolic risk features and prevalence of coronary artery disease (CAD).

**Aim:**

To investigate the association of *eNOS* Glu298Asp gene polymorphism, cardiometabolic profile, obstructive CAD and inducible myocardial ischemia in patients with suspected stable CAD.

**Methods:**

A total of 506 patients (314 males; mean age 62 ± 9 years) referred for suspected CAD was enrolled. Among these, 325 patients underwent stress ECG or cardiac imaging to assess the presence of inducible myocardial ischemia and 436 patients underwent non-invasive computerized tomography or invasive coronary angiography to assess the presence of obstructive CAD. Clinical characteristics and blood samples were collected for each patient.

**Results:**

In the whole population, 49.6% of patients were homozygous for the Glu298 genotype (Glu/Glu), 40.9% heterozygotes (Glu/Asp) and 9.5% homozygous for the 298Asp genotype (Asp/Asp). Obstructive CAD was documented in 178/436 (40.8%) patients undergoing coronary angiography while myocardial ischemia in 160/325 (49.2%) patients undergoing stress testing. Patients with *eNOS* Asp genotype (Glu/Asp + Asp/Asp) had no significant differences in clinical risk factors and in circulating markers. Independent predictors of obstructive CAD were age, gender, obesity, and low HDL-C. Independent predictors of myocardial ischemia were gender, obesity, low HDL-C and Asp genotype. In the subpopulation in which both stress tests and coronary angiography were performed, the Asp genotype remained associated with increased myocardial ischemia risk after adjustment for obstructive CAD.

**Conclusion:**

In this population, low-HDL cholesterol was the only cardiometabolic risk determinant of obstructive CAD. The *eNOS* Glu298Asp gene polymorphism was significantly associated with inducible myocardial ischemia independently of other risk factors and presence of obstructive CAD.

**Supplementary Information:**

The online version contains supplementary material available at 10.1186/s12872-024-03846-7.

## Introduction

Coronary artery disease (CAD) is a multifactorial disease with a complex pathogenesis, which manifests clinically with myocardial ischemia syndromes and continues to be the first cause of death worldwide [1, 2], especially in low-to-middle-income countries. The pathogenesis of CAD includes the interaction of multiple non-modifiable and modifiable risk factors associated with a progressive atherosclerotic process leading to obstructive disease [3]. Ischemic manifestations are generally a consequence of obstructive CAD but are also dependent on functional abnormalities of coronary vessels [4].

Growing evidence has consistently indicated endothelium, the main actor of vascular function, as a target of many cardio-metabolic conditions associated with atherogenesis and ultimately with CAD [5]. The endothelium is a highly dynamic cell layer involved in a multitude of physiologic processes ranging from the regulation of vascular tone to the homeostasis of systemic metabolism. The endothelial function is mainly guaranteed by the production of vasoactive molecules, particularly nitric oxide (NO) by endothelial nitric oxide synthase (*eNOS*), which plays an atheroprotective and vasodilatory role [6].

Experimental and clinical studies have suggested endothelium as an independent determinant of both vascular dysfunction and cardiometabolic deregulation. Total eNOS knockout mice (eNOS-/-), develop either coronary vascular dysfunction and hypertension, insulin resistance, hyperlipidemia [7–9]. Interestingly, also mice with a partial eNOS gene deletion (eNOS ±) demonstrate coronary vascular dysfunction while developing hypertension and overt insulin resistance when fed with a high-fat diet [8, 9].

In humans, single-nucleotide polymorphisms (SNPs) of the *eNOS*-encoding gene have been related with both CAD [10] and systemic metabolic abnormalities [11, 12]. In particular, its Glu298Asp variant has been associated with CAD predisposition [11] and with myocardial ischemia also independently of the presence of obstructive CAD [13]. Multiple *eNOS* gene variants have also been associated with the cardio-metabolic features of the metabolic syndrome (MeS) in the general population [14] and in patients with cardiovascular disease [15]. This is relevant since the MeS and its components have received recently particular attention as determinant of residual risk of CAD, i.e. the risk persisting despite current medical treatment [16, 17].

Despite these multiple evidences, there are not available studies in current patient populations able to clarify whether genetically determined eNOS abnormalities and cardiometabolic risk determinants have an independent or synergistic role in CAD and myocardial ischemia manifestations. In the frame of these premises, this study aimed to investigate the specific association of the *eNOS* Glu298Asp polymorphism and cardiometabolic risk factors with obstructive CAD and inducible myocardial ischemia in a population of patients with suspected stable coronary disease.

## Materials and methods

### Study design, patient population, and ethical approval

**Fig. 1:**
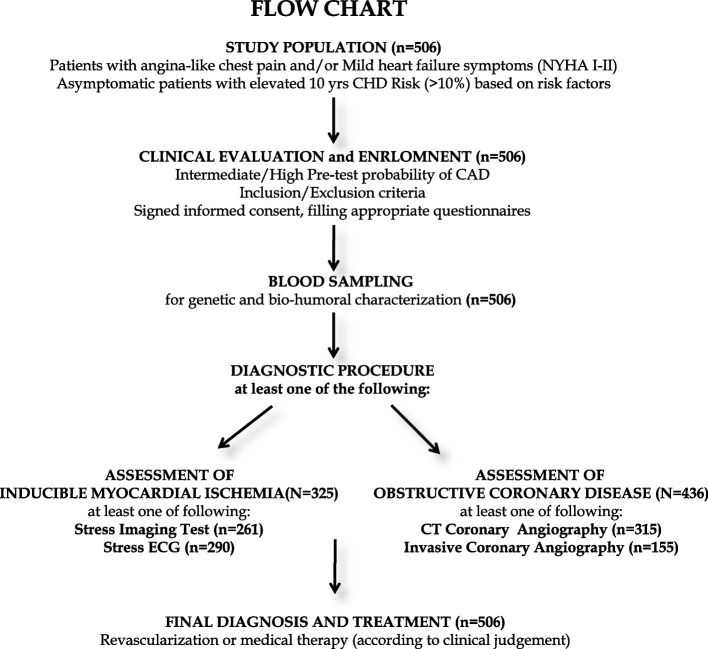
Flow chart of the BIOGEN-CARE Tuscan Region Italian Study

A total of 506 consecutive patients (314 males; mean age 62 ± 9 years) referred for suspected CAD within the BIOGEN-CARE Tuscan Region Italian Study were enrolled in six cardiology units. Among these, 325 patients underwent cardiac stress test (including stress ECG and/or cardiac imaging) to assess the presence of inducible myocardial ischemia and 436 patients underwent coronary study (including non-invasive computerized tomography and/or invasive coronary angiography) to assess the presence of obstructive CAD (> 50% stenosis in at least one major coronary vessel). In 255 patients both cardiac stress tests and coronary angiography were performed. The study flow chart is presented in Fig. 1.

The study was conducted according to the Declaration of Helsinki and its later amendments. The protocol was approved by the Coordinating Center Ethical Committee and all local Ethical Committees. All patients included in the study signed a written informed consent.

### Clinical and laboratory characterization

For each patient, clinical and demographic characteristics including age, gender, smoking, family history of CAD, left ventricular ejection fraction (LVEF %) and medical therapy were recorded at enrollment before any diagnostic testing. Blood samples were collected from each patient for genotyping and measurements of clinical lipid and glucose parameters. Glucose, insulin, Total-cholesterol (Total-C), triglycerides (TG), low-density lipoprotein cholesterol (LDL-C), high-density lipoprotein cholesterol (HDL-C), and Apolipoprotein A1 (ApoA1) levels (mg/dL) were evaluated using standard clinical chemistry laboratory methods. The HOMA index was calculated from the formula: HOMA index = fasting serum insulin (μU/ml) x fasting plasma glucose (FPG) (mmol/l)/22.5. The TG/HDL-C ratio was calculated as TG level divided by HDL-C levels.

The recorded non modifiable cardiovascular disease (CVD) risk factors included age, gender, family history of CVD. The modifiable CVD risk factors were defined, according to current international standards for CVD risk assessment [3] and for the definition of the MeS [18], based on patients’ clinical records plus annotated treatment and/or, when appropriate, on available clinical and biohumoral measurements. Definitions for modifiable CVD risk factors were: high systolic blood pressure (SBP) (hypertension under treatment or SBP > 130/85 mmHg), high LDL-C (hypercholesterolemia under treatment with statins or other lipid lowering drugs, or LDL-C > 115 mg/dL), previous or current tobacco smoking, high body mass index (BMI > 30 kg/m2), diabetes (treatment with hypoglycemic agents or insulin or FPG > 126 mg/dL), high FPG (diabetes or FPG > 100 mg/dL), high TG (> 150 mg/dL) and low HDL-C (< 40 mg/dL in males and < 50 mg/dL in females). The presence of MeS was defined by the presence in each patient of at least 3 of the following risk factors: high SBP, high BMI as a proxy for obesity, high FPG, high TG and low HDL-C [19].

### Genetic analysis

DNA was extracted from peripheral blood by using QIAamp DNA Blood Mini Kit (Qiagen, CA, USA) according to manufacturer’s instructions. The *eNOS* Glu298Asp polymorphism was determined by a standard polymerase chain reaction (PCR) amplification using the HotStarTaq Master Mix kit (QIAGEN Inc., Valencia, CA, USA) as follows: a first step of DNA denaturation at 95 ◦C for 15 min, 32 cycles at 94 ◦C for 30 s, 58 ◦C for 30 s and 72 ◦C for 1 min, and a final extension step at 72 ◦C for 10 min. Primers (F5’ -CATGAGGCTCAGCCCCAGAAC-3’ and R5’—AGTCAATCCCTTTGGTGCTCAC-3’) were selected from the eNOS Ensemble sequence database (Ensembl:ENSG00000164867) and designed using the free web-based application Primer3Plus (https://www.bioinformatics.nl/cgi-bin/primer3plus/primer3plus.cgi (accessed on 10 September 2021).

### Statistical analysis

Continuous variables are presented as mean ± standard deviation (SD), categorical variables as relative percentages. Continuous data were first checked for normality (and ln transformed if required) before using parametric tests. Differences between two groups were compared by χ^2^ test for categorical variables or by two-tailed tests, unpaired Student’s t-test, for quantitative variables. Comparison of 3 means was performed by ANOVA, followed by Bonferroni post hoc test for comparison of any 2 groups. Multivariate linear regression was used to estimate the effect of cardiometabolic risk factors including age, male sex, family history of CAD, high SBP (as defined for MeS), high LDL-c (> 115mg/dL), smoking, BMI > 30 kg/m2, high FPG (as defined for MeS), low HDL-c (as defined for MeS), and high triglycerides (as defined for MeS), and *eNOS* Glu298Asp polymorphism (according to the dominant model Glu/Glu vs Asp) on obstructive CAD and/or inducible myocardial ischemia. A multivariable model was developed, including all variables with a *p* value ≤ 0.1 at univariable analysis, and then using backward and forward stepwise selections to build-up the final model. To not overfit the model with related variables, the variables were tested for collinearity. A *p* value < 0.05 was considered statistically significant. All statistical analyses were completed using Stata/SE 13.1 and SPSS Version 24.

## Results

### Clinical risk factors, metabolic profiles and eNOS genotype

The clinical risk factors, the metabolic characteristics and the eNOS genotype distribution of the whole study population and of groups defined according to the presence/absence of obstructive CAD or presence/absence of inducible myocardial ischemia are shown in Table 1.

**Table 1:** Comparison of clinical characteristics, modifiable risk factors, laboratory clinical-chemistry data and eNOS genotype distribution among patient groups defined by absence/presence of obstructive CAD and absence/presence of inducible myocardial ischemia

	**Whole** **population** ***n* = 506**	**ObstrCAD-NO** ***n* = 258**	**ObstrCAD-YES** ***n* = 178**	***p*-value**	**InducibleMI-NO** ***n* = 160**	**InducibleMI-YES** ***n* = 165**	***p*-value**
***Clinical characteristics***							
* Age, years*	62.0 ± 9.2	60.3 ± 8.4	64.6 ± 8.6	< 0.0001	60.5 ± 9.9	63.9 ± 9.3	0.0006
* Male gender*	62	52	81	< 0.0001	54	68	0.01
* Family history of CAD*	49	51	42	ns	57	46	ns
* LVEF*	60.7 ± 5.1	61.9 ± 4.9	59.5 ± 5.1	0.0001	61.1 ± 4.9	60.4 ± 5.2	ns
***Symptoms***							
* Typical Angina*	40	33	53	< 0.0001	41	51	ns
***Medications***							
* Antihypertensives and Anti-ischemics*	69	69	70	ns	68	65	ns
* Aspirin*	44	46	43	ns	48	48	ns
* Statins*	38	39	37	ns	26	28	ns
* Anti diabetic therapy*	16	18	16	ns	16	19	ns
***Modifiable Risk factors***							
* High SBP*^a^	64	63	69	ns	62	70	ns
* High LDL-C*	48	51	43	ns	61	48	0.02
* Smoking*	20	17	24	ns	19	22	ns
* High BMI*^a^	20	23	16	ns	25	11	0.0009
* High FPG*^a^	55	51	60	ns	56	50	ns
* Low HDL-C*^a^	28	22	40	< 0.0001	18	28	0.01
* High TG*^a^	25	22	29	ns	24	28	ns
* Metabolic Syndrome*	30	26	36	0.04	30	27	ns
***Laboratory clinical-chemistry data***							
* Total-C, mg/dL*	190.0 ± 48.1	196.7 ± 48.5	177.1 ± 47.5	< 0.0001	204.8 ± 43.5	190.5 ± 44.5	0.003
* LDL-C, mg/dL*	113 ± 39	117 ± 40	107 ± 38	0.0009	124 ± 35	114 ± 41	0.007
* HDL-C, mg/dL*	52.9 ± 16.9	57.5 ± 18.2	45.4 ± 13.3	< 0.0001	56.9 ± 16.7	53.0 ± 18.1	0.009
* TG, mg/dL*	118.0 ± 64.0	112.3 ± 57.5	123.2 ± 65.4	ns	119.5 ± 66.8	122.3 ± 67.0	ns
* ApoA1, mg/dL*	132 ± 41	142 ± 44	139 ± 37	< 0.0001	134 ± 41	132 ± 42	ns
* TG/HDL-C*	2.6 ± .1.8	2.3 ± 1.7	3.0 ± 1.9	< 0.0001	2.4 ± 1.7	2.7 ± 1.9	ns
* Glucose, mg/dL*	105.0 ± 29.1	103.7 ± 24.8	107.3 ± 32.0	ns	108.8 ± 31.3	103.7 ± 33.7	ns
* Insulin, μUI/mL*	9.8 ± 9.7	9.6 ± 8.2	10.5 ± 11.0	ns	10.4 ± 10.0	9.4 ± 8.9	0.05
* HOMA Index*	2.8 ± 3.8	2.6 ± 2.4	2.8 ± 3.6	ns	3.1 ± 4.4	2.6 ± 2.8	0.02
***Genotype distribution***							
* Glu/Glu*	50	48	50	ns	59	42	0.01
* Glu/Asp*	41	43	39		33	48	
* Asp/Asp*	9	9	11		8	10	
* Glu/Glu*	50	47	50	ns	59	42	0.003
* Asp*	50	53	50		41	58	

Continuous variables are presented as mean ± standard deviation, categorical variables as (%)

*ObsCAD* obstructive coronary artery disease, *inducibleMI* inducible myocardial ischemia, *high SBP (SBP* = *systolic blood pressure)* SBP ≥ 130/85 mmHg or antihypertensive medication, *Total-C* (C = cholesterol), *high LDL-C (LDL-C* = *LDL cholesterol)* LDL-C > 115mg/dL, *high BMI (BMI* = *body mass index)* BMI > 30 kg/m2, *high FPG (FPG* = *fasting plasma glucose)* high FPG diabetes or FPG > 100 mg/dL, *low HDL-C (HDL-C* = *HDL cholesterol)* HDL-C < 50 mg/dl in women and < 40 mg/dl in men or specific treatment for this lipid abnormality, *high TG (TG* = *triglycerides)* TG ≥ 150 mg/dl or specific treatment for this lipid abnormality, *ApoA1* Apolipoprotein 1

^a^component of metabolic syndrome

Obstructive CAD was diagnosed in 178/436 (41%) patients undergoing coronary study. Patients with obstructive CAD were older, mainly males, had lower LVEF%, and more frequently referred typical angina. Patients with obstructive CAD, as compared with those without, had a similar traditional risk profile and received similar treatments at enrollment, but had a higher prevalence of low HDL-C (40 vs 22%, *p* < 0.001) and MeS (36 vs 26%, *p* = 0.04). Moreover, patients with obstructive CAD had lower levels of Total-C, LDL-C, HDL-C and ApoA1 as well as a higher TG/HDL-C ratio than patients without obstructive CAD.

Myocardial ischemia was documented in 160/325 (49.2%) patients undergoing stress testing. The risk profile of patients with inducible myocardial ischemia differed from that of patients without for older age, more frequent male gender and low HDL-C while high LDL-C and obesity were less frequent. They had lower levels of Total-C, LDL-C, HDL-C, as well as a lower levels of insulin and a lower HOMA index.

The genotype analysis in the whole population yielded 251 (50%) patients homozygous for the G894 allele (GG, Glu298Glu, Glu/Glu), 207 (41%) heterozygotes (GT, Glu298Asp, Glu/Asp), and 48 (9%) homozygous for the T894 allele (TT, Asp298Asp, Asp/Asp). The prevalence of the *eNOS* alleles satisfied the Hardy–Weinberg equilibrium law (χ^2^ = 0.31, *p* = 0.58). No difference in the genotype distribution of *eNOS* Glu298Asp variant was observed between patients with or without obstructive CAD. On the other hand, the *eNOS* Asp genotype was significantly more frequent in patients with inducible myocardial ischemia than in patients without (Table 1).

Between the clinical characteristics, only gender was significantly different in patients with different genotype with a male predominance in individuals with the mutant Asp genotype (Glu/Asp and Asp/Asp). No statistically significant differences in cardiometabolic risk factors prevalence and treatments were observed between groups. At laboratory analysis, patients with the mutant genotype showed lower levels of HDL-C, and ApoA1 as well as a higher TG/HDL-C ratio (Supplementary Table 1).

A multivariate logistic regression analysis was performed to identify risk factors (including non-modifiable and modifiable determinants) associated with obstructive CAD or inducible myocardial ischemia (Table 2).

**Table 2:** Predictors of obstructive CAD or inducible myocardial ischemia at univariate and multivariate analyses by logistic regression analysis

	***Obstructive CAD***		***Inducible Myocardial Ischemia***	
	**Univariate analysis** **OR (CI 95%), *p*-value**	**Multivariate analysis** **OR (CI 95%), *p*-value**	**Univariate analysis** **OR (CI 95%), *p*-value**	**Multivariate analysis** **OR (CI 95%), *p*-value**
**Age**	1.1 (1.0–1.1), *p* < 0.0001	1.1(1.0–1.11), *p* < 0.0001	1.1(1.0–1.1), *p* < 0.0001	ns
**Male sex**	4.0(2.6–6.3), *p* < 0.0001	4.8(2.9–8.1), *p* < 0.0001	4.0(2.6–6.3), *p* < 0.0001	1.9(1.1–3.1), *p* = 0.01
**Family History of CAD**	0.7(0.5–1.1), *p* = 0.09	ns	0.7(0.5–1.1), *p* = 0.09	ns
**High SBP**^a^	1.3(0.8–1.9), *p* = 0.1	ns	1.4(0.9–2.3), *p* = 0.1	ns
**High LDL-C**	0.7(0.5–1.1), *p* = 0.09	ns	0.6(0.4–0.9), *p* = 0.02	ns
**Smoking**	1.5(0.9–2.5), *p* = 0.07	ns	1.2(0.7–2.1), *p* = 0.5	-
**High BMI**^a^	0.6(0.4–1.0), *p* = 0.07	0.5(0.3–0.9), *p* = 0.03	0.4(0.2–0.7), *p* = 0.01	0.3(0.2–0.6), *p* = 0.0002
**High FPG**^a^	1.4(0.9–2.1), *p* = 0.06	ns	0.8(0.5–1.2), *p* = 0.3	-
**Low HDL-C**^a^	2.5(1.6–3.7), *p* < 0.0001	2.8(1.7–4.7), *p* = < 0.0001	1.9(1.1–3.2), *p* = 0.01	2.0(1.1–3.6), *p* = 0.04
**High TG**^a^	1.5(0.9–2.2), *p* = 0.09	ns	1.3(0.8–2.1), *p* = 0.3	-
**eNOS Asp genotype**	0.9(0.6–1.3), *p* = 0.6	-	1.9(1.2–3.0), *p* = 0.003	2.1(1.3–3.4), *p* = 0.003

*ns* not significant, *CAD* coronary artery disease, *high SBP (SBP* = *systolic blood pressure)* SBP ≥ 130/85 mmHg or antihypertensive medication, *high LDL-C (LDL-C* = *LDL cholesterol)* LDL-C > 115 mg/dL, *high BMI (BMI* = *body mass index)* BMI > 30 kg/m2, *high FPG (FPG* = *fasting plasma glucose)* high FPG: diabetes or FPG > 100 mg/dL, *low HDL-C (HDL-C* = *HDL cholesterol)* HDL-C < 50 mg/dl in women and < 40 mg/dl in men or specific treatment for this lipid abnormality, *high TG (TG* = *triglycerides)* TG ≥ 150 mg/dl or specific treatment for this lipid abnormality

^a^component of metabolic syndrome

Among non-modifiable risk factors, older age and male sex were independent predictors of obstructive CAD. The eNOS genotype was not associated with obstructive disease. Among modifiable risk factors, the MeS was not an independent determinant of obstructive CAD (Supplementary Table 2). Among MeS components only low levels of HDL-C were independently associated with obstructive CAD while obesity was inversely associated.

At multivariate logistic regression analysis for the prediction of inducible myocardial ischemia, the *eNOS* Asp genotype was a strong independent determinant of myocardial ischemia together with gender and low HDL-C levels while obesity remained inversely associated (Table 2). The MeS was not an independent determinant of inducible myocardial ischemia (Supplementary Table 2).

In the subgroup of patients who underwent both cardiac stress tests and coronary angiography (*n* = 255) the eNOS 298Asp genotype remained associated with an increased prevalence of inducible myocardial ischemia even after adjustment for the presence of obstructive CAD (OR 2.1; CI 1.2–3.7, *p* = 0.01) (Table 3). The clinical characteristics, risk factors and metabolic profiles were compared in the two extreme clinical presentations of this subgroup of patients (Supplementary Table 3). Patients having both obstructive CAD and inducible myocardial ischemia (CAD/MI, *n* = 84) were older, mainly males, with more frequent typical angina and with significantly reduced LVEF% compared with patients having neither obstructive CAD nor inducible myocardial ischemia (noCAD/noMI, *n* = 87). Regarding the cardiometabolic profile, CAD/MI patients had lower prevalence of obesity (12 vs 31%, *p* = 0.002) and of high LDL-C (39 vs 61%, *p* = 0.003) but a higher prevalence of low HDL-C (39 vs 10%, *p* < 0.001) and high TG (36 vs 22%, *p* = 0.014) compared to noCAD/noMI patients. Differences in laboratory data mirrored those in the cardiometabolic profile as shown in the whole population of patients with or without obstructive CAD (Table 1). The *eNOS* Asp genotype was more prevalent in CAD/MI compared to noCAD/noMI patients (58% vs 42%, *p* = 0.03).

**Table 3:** Predictors of inducible myocardial ischemia at univariate and multivariate analyses by logistic regression analysis in patients who underwent both cardiac stress tests and coronary angiography (*n* = 255)

	**Univariate analysis** **OR (CI 95%), *p*-value**	**Multivariate analysis** **OR (CI 95%), *p*-value**
Age	1.0(1.0–1.1), *p* = 0.04	ns
Male sex	2.1(1.2–3.4), *p* = 0.008	ns
Family History of CAD	0.9(0.6–1.6), *p* = 0.9	-
High SBP^a^	1.3(0.8–2.2), *p* = 0.4	-
High LDL-C	0.7(0.4–1.1), *p* = 0.1	-
Smoking	1.1(0.6–2.0), *p* = 0.8	-
High BMI^a^	0.4(0.2–0.4), *p* = 0.001	0.3(0.1–0.5), *p* = 0.0005
High FPG^a^	0.8(0.5–1.2), *p* = 0.3	-
Low HDL-C^a^	2.3(1.3–4.3), *p* = 0.007	ns
High TG^a^	1.5(0.9–2.7), *p* = 0.1	-
eNOS Asp genotype	1.9(1.2–3.0), *p* = 0.01	2.1(1.2–3. 7), *p* = 0.01
Obstructive CAD	4.3(2.6–7.4), *p* < 0.0001	3.1(1.67–5.8), *p* = 0.0004

*ns* not significant, *CAD* coronary artery disease, *high SBP (SBP* = *systolic blood pressure)* SBP ≥ 130/85 mmHg or antihypertensive medication, *high LDL-C (LDL-C* = *LDL cholesterol)* LDL-C > 115 mg/dL, *high BMI (BMI* = *body mass index)* BMI > 30 kg/m2, *high FPG (FPG* = *fasting plasma glucose)* high FPG: diabetes or FPG > 100 mg/dL, *low HDL-C (HDL-C* = *HDL cholesterol)* HDL-C < 50 mg/dl in women and < 40 mg/dl in men or specific treatment for this lipid abnormality, *high TG (TG* = *triglycerides)* TG ≥ 150 mg/dl or specific treatment for this lipid abnormality

^a^component of metabolic syndrome

## Discussion

In this population of patients with suspected stable CAD, low HDL-C was the only cardiometabolic risk determinant independently associated with both obstructive disease and inducible myocardial ischemia. The *eNOS* Asp genotype was not associated with obstructive CAD but was a significant determinant of inducible myocardial ischemia independently of other risk factors, even after adjustment for presence of obstructive CAD. Obesity resulted inversely associated with both obstructive CAD and inducible myocardial ischemia.

Evidence shows that each component of the standard lipid profile including total cholesterol, HDL-C, LDL-C, and triglycerides are markers of cardiovascular risk [20] although the attention has been largely focused on total cholesterol and LDL-C levels since their substantial reduction is able to decrease the occurrence of cardiovascular events and the progression of coronary atherosclerosis [20, 21]. Nevertheless, more recently, a number of studies have shown an association between the co-occurrence of high TG and low HDL-C with a specific cardiometabolic profile, also referred to the pathophysiologic condition known as atherogenic dyslipidemia, strictly linked to cardiovascular atherosclerotic risk, but independent of LDL-C and LDL-C lowering therapies [17, 22].

More in general, low HDL-C and high TG are key components of the MeS that is defined by the presence of at least three out of five clinical features also including obesity, hypertension and hyperglycemia. The MeS has been related with increased risk of developing cardiovascular disease [19].

One of the major results in our population was that the MeS was not an independent determinant of obstructive CAD while low HDL-C was its only component that increased the risk of obstructive disease.

This finding expands the discussion around the role of HDL-C in contributing to the global atherosclerotic risk. The proposed effect of low HDL-C in predisposing to obstructive CAD is due to the anti-atherosclerotic properties of HDL mainly involving the so called “reverse cholesterol transport” mechanism [23], by which excess cholesterol is removed from the vessels wall and is delivered to the liver where it is metabolized. The efficiency of this mechanism is not only related with the abundance of HDL-C particles but also with their functionality. For example, there is recent evidence that high levels of TG rich lipoproteins cause not only a decrease in HDL lipoproteins, due to enhanced catabolism, but also some structural changes [24]. The alteration of HDL composition is able to decrease the anti-atherosclerotic properties of these particles involving not only their contribution to the reverse cholesterol transport but also their anti-inflammatory actions [25]. In the present study, high levels of TG were not per-se independent determinants of obstructive CAD risk but patients with obstructive CAD had significantly increased TG/HDL-C ratio as documented in other studies [17]. While the potential role of TG rich lipoproteins has been recently posed to great attention as a new direct determinant of residual atherosclerotic risk, the independent role of HDL-C is debated also due to the apparent inefficacy of treatments developed so far to increase HDL-C levels [26]. In our, as in previous studies, there are confounding factors which could influence HDL-C levels and their possible pathogenetic effects such as the use of lipid modifying medications. This area definitely needs further investigations.

Another important result of the present study was that low levels of HDL-C were predictors of both obstructive CAD and inducible MI together with the *eNOS* 298Asp SNP, suggesting a pathophysiologic interaction between the properties of HDL and a genetically determined primary endothelial dysfunction such that associated with *eNOS* Glu298Asp polymorphism [27]. While the association of low HDL-C with inducible myocardial ischemia in this population appeared mainly driven by its association with obstructive CAD, the link between the *eNOS* genetic variant and myocardila ischemia resulted independent.

Classically, the substrate of ischemic heart disease is mainly attributed to the presence of obstructive CAD although growing evidence demonstrates that this is not the only determining factor. Indeed, vessel obstruction is not always present in patients with angina [27, 28] and, on the other hand, myocardial ischemia may affect patients without obstructive CAD. Myocardial ischemia is a complex process involving more coronary districts from larger epicardial arteries to coronary microvessels. A “primitive” (genetic) endothelial dysfunction might represent a shared pathophysiological mechanism that, affecting both large and micro-vessels may determine myocardial ischemia [27]. The endothelium is a highly active metabolic and endocrine organ producing a multitude of different molecules, including NO which exerts a main role in both vascular and microvascular homeostasis [29].

In endothelium, NO is mainly produced by eNOS starting from L-arginine, multiple cofactors, and prosthetic groups. Nitric oxide plays an atheroprotective effect able to counteract harmful conditions (oxidative stress, inflammation, and platelet dysfunction) that trigger vascular damage in large epicardial arteries but, it has also a major role at the microcirculation level in the regulation of adequate coronary blood to be supplied to the myocardium [30].

We previously showed that mice with a partial or total deletion of eNOS gene had an impairment of coronary vasodilating capability (as documented by higher coronary resistance and lower percent decrease during Ach infusion) compared with wild types [9].

In humans, genetic variants of *eNOS* gene have been associated with cardiovascular disease including ischemic heart disease and its clinical manifestation. In particular, the point mutation G894T in the coding sequence of the *eNOS* that modifies the glutamic acid in aspartic acid at codon 298 (Glu298Asp) modifies the primary structure of the protein and has the potential to alter one or more functional properties of the enzyme. Two different studies have shown the eNOS protein containing Asp at position 298 is subject to selective proteolytic cleavage in endothelial cells and vascular tissues leading to a decreased NO synthesis [31, 32]. Two other reports showed contrasting results [33, 34]. However, it has been demonstrated that the *eNOS* G894T SNP affects the enzyme localization to caveolar membrane harming the eNOS signaling in response to shear stress [35]. In cardiovascular genetic association studies, the Glu298Asp variant has been associated with the development of myocardial infarction in very young individuals, whose coronary arteries are characterized by a very small atheromatic burden [36]. Additionally, this genetic variant was an independent risk factor for early STEMI presentation in young (under 45 years) patients, emphasizing the pivotal role of genetic susceptibility to endothelial dysfunction [37]. Additionally, the Glu298Asp polymorphism in the *eNOS* gene has been associated with coronary spasm in patients with variant angina, independently of the degree of atherosclerotic burden [38]. Notably, it had been shown that the T(-786)C promoter polymorphism and its interaction with exon 7 Glu298Asp affect endothelium-dependent vasodilation in human forearm microcirculation [39]. As a matter of fact, similarly to our present results, in a meta-analysis study, Luo et al. showed that the *eNOS* Glu298Asp polymorphism was associated with myocardial ischemia, also when corrected for the traditional cardiovascular risk factors [40]. More recently, in a population of patients with suspected acute coronary syndrome, the *eNOS* 298Asp SNP was an independent predictor of ischemic heart disease and clinical presentation of acute coronary syndrome [13]. The findings of our study confirm and expand all these previous reports showing a strong association of endothelial genetic predisposition with inducible myocardial ischemia independently of the presence of epicardial arteries obstructive disease.

Finally, in our population, obesity resulted inversely associated with both obstructive CAD and inducible myocardial ischemia. Obesity, a complex, multifactorial condition characterized by abnormal or excessive accumulation of body fats is increasing progressively in recent decades worldwide and it is one of the most significant global health challenges [41]. It is generally recognized as a risk factor for CAD mainly through its influence on the development and severity of comorbidities including hypertension, dyslipidemia, and glucose intolerance or diabetes [42]. Nevertheless, once CAD has developed, overweight or obese individuals have lower mortality than normal-weight people, the so-called “obesity paradox” [43]. The mechanism of the obesity paradox remains mainly unknown although it has been related with variable individual production of adipokines able to potentially neutralize the action of circulating pro-inflammatory cytokines involved in the atherosclerotic process [44]. In addition, clinical and experimental evidence indicates that obesity may be an independent risk factor for coronary microvascular dysfunction, possibly contributing to inducible myocardial ischemia, [45]. Cortigiani et al. recently demonstrated that obesity exerted a “paradoxical” protective effect in patients with stress-induced ischemia and/ or coronary microvascular dysfunction in a population of patients with known or suspected CAD [46]. In the present study obesity was not a significant predictor of obstructive CAD and/or inducible myocardial ischemia and our results suggest a possible protective role at least in this population.

### Clinical implication

From a clinical point of view, the results of this study, whether confirmed in larger populations, highlight the possible role of endothelial genetic dysfunction in predisposing to inducible myocardial ischemia independently of the presence of epicardial arteries obstructive atherosclerotic disease. The underlying genetic and molecular pathways beyond myocardial ischemia in the absence of obstructive CAD are complex and deserve continuing mechanistic research. The study of genetically induced endothelial dysfunction may represent a major aspect in the comprehension of pathophysiological mechanisms governing myocardial ischemia independently (or jointly) to traditional risk factors. Interestingly in the present study we have shown that low HDL-C is among the modifiable cardiometabolic risk determinant the only one which is independently associated with both obstructive CAD and inducible myocardial ischemia. The possible synergistic actions between endothelial genetic dysfunction and HDL in determining the pathogenesis and the clinical manifestations of ischemic heart disease remain to be investigated. Confirming the major impact of a defective *eNOS* gene on myocardial ischemia induction could also pave the way for the research of novel new targeted drug strategies. Moreover, the identification of specific genetic and cardiometabolic profiles will allow recognizing patients at high risk for obstructive CAD and myocardial ischemia which might be fundamental for their prognostic stratification and correct management.

### Study limitations

This study has several limitations. The study population was relatively small and included mainly patients from the Tuscany Region of Italy and, definitely, with the same Caucasian ethnicity. There are dissimilarities in the frequencies of *eNOS* Glu298Asp variants in different races thus, it is unknown whether our findings may be extended to a different population of patients with similar clinical features. Then, we investigated only a single SNP of the *eNOS* gene although previous studies suggested that there is linkage disequilibrium among the most clinically relevant *eNOS* SNPs. It is conceivable that other SNPs in eNOS gene as well as in other genes affecting endothelial function including also genes linked to lipoprotein metabolism can interact each other’s. Moreover, our population lacks information on waist circumference, which is a critical component of the MeS definition of central obesity. Finally, our patients were enrolled based on referral for suspected stable CAD and patients with previous disease were excluded. Accordingly, the present population is at relatively low risk and these results cannot be extended to populations with known CAD or at higher risk.

## Conclusions

**Fig. 2:**
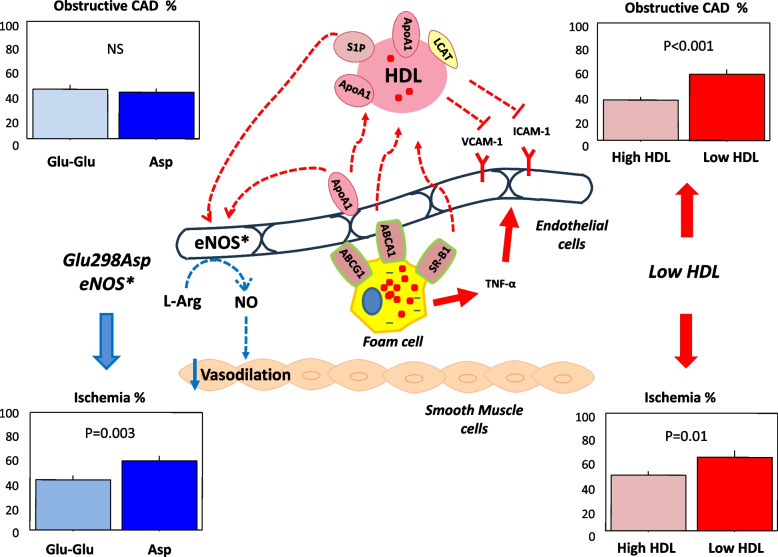
Schematic representation of the possible mechanisms underlying the association of eNOS Glu298Asp polymorphism and low levels of HDL with inducible myocardial ischemia and obstructive CAD. The endothelial cells produce a multitude of different molecules, including nitric oxide (NO) which is mainly generated by endothelial nitric oxide synthase (eNOS) starting from L-arginine. The eNOS Glu298Asp polymorphism has been shown to modify the primary structure of the enzyme leading to a decreased NO synthesis causing reduced macrovascular and microvascular vasodilation (*Tesauro M *et al*. Proc Natl Acad Sci U S A. 2000; Persu A *et al*. Hum Mol Genet 2002*) which may trigger inducible myocardial ischemia. Reduced NO availability also limits its antiatherogenic properties, i.e., its capability to counteract conditions such as oxidative stress, and inflammation. On the other hand, an alteration of either the abundance and/or composition of HDL, is able to decrease the anti-atherosclerotic properties of these particles involving not only their contribution to the reverse cholesterol transport (RCT) but also their anti-inflammatory actions. HDL is involved in RCT by scavenging cholesterol from foam cells in atherosclerotic plaque. Briefly, ApoA-I initiates cholesterol efflux by binding to adenosine triphosphate-binding cassette transporter A1 (ABCA1), with further uptake through the ATP-binding cassette transporter G1 (ABCG1) and scavenger receptor class B type I (SR-BI). Cholesterol, esterified by lecithin-cholesterol acyl transferase (LCAT), is included into the lipid core of mature HDL, and then transported to the liver for excretion. HDL also promotes endothelial function by stimulating eNOS via shingosine-1-phosphate (S1P), and ApoA-I (*Vecoli C et el. J Cell Biochem 2011*). Moreover, HDL inhibits the expression of recruitment factors for inflammatory cells such as intercellular adhesion molecule-1 (ICAM-1), and vascular cell adhesion molecule (VCAM-1), stimulated by tumor necrosis factor-α (TNF-α) which is released by foam cells. Dotted Arrows denote reduced effects while dotted T bars denote reduced inhibitory effects

In patients with suspected stable CAD, the *eNOS* Glu298Asp gene polymorphism was a risk factor for inducible myocardial ischemia independent of the presence of obstructive coronary lesions and of other established risk determinants. In this population, among additional cardiometabolic risk factors, only low HDL-C was independently associated with obstructive CAD and, together with the *eNOS* Glu298Asp gene polymorphism, with inducible myocardial ischemia. In Fig. 2 a diagrammatic representation of possible mechanisms underlying the association of eNOS Glu298Asp polymorphism and low levels of HDL-C with inducible myocardial ischemia and obstructive CAD is presented. Further studies are needed to explore the role of *eNOS* genetic variants and their interaction with a specific cardiometabolic profile in ischemic heart disease.

### Supplementary Information

**Supplementary Material 1.**

## Data Availability

The data and materials underlying this article will be shared on reasonable request to the corresponding authors.

## Abbreviations

CAD: Coronary artery disease
MI: Myocardial ischemia
HDL-C: HDL cholesterol
eNOS: Endothelial nitric oxide synthase
SBP: Systolic blood pressure
Total-C: Total cholesterol
LDL-C: LDL cholesterol
BMI: Body mass index
FPG: Fasting plasma glucose;
TG: Triglycerides
ApoA1: Apolipoprotein 1
