# Supplementary material for: Low HDL cholesterol and the eNOS Glu298Asp polymorphism are associated with inducible myocardial ischemia in patients with suspected stable coronary artery disease
Source: BMC Cardiovasc Disord. 2024 Mar 22;24:176. doi: 10.1186/s12872-024-03846-7 (PMC10958845; doi:10.1186/s12872-024-03846-7)
Supplement: Supplementary file 1 — Supplementary Material 1. [file 12872_2024_3846_MOESM1_ESM.pdf]

Table 1 Suppl mat: *Comparison of clinical characteristics, modifiable risk factors and clinical-chemistry laboratory data among patient groups defined by eNOS genotypes.*

**Clinical characteristics**	**Glu/Glu genotype n=251**	**Glu/Asp genotype n=207**	**Asp/Asp genotype n=48**	**p-value**	**Glu/Glu genotype n=251**	**Asp genotype n=255**	**p-value**
Age, years	62.1±8.7	62.2±9.4	60.0±10.1	ns	62.1±8.8	61.8±9.6	ns
Male gender	56	66	75	0.01	56	68	0.007
Family history of CAD	47	52	50	ns	47	51	ns
LVEF %	61.0±5.1	60.4±5.4	61.1±5.7	ns	61.0±5.1	60.5±5.0	ns
Symptoms							
Typical angina	41	39	39	ns	41	39	ns
Medications							
Antihypertensives and Anti-ischemics	66	73	71	ns	66	73	ns
Aspirin	42	46	50	ns	42	47	ns
Statins	33	38	50	ns	33	40	ns
Anti diabetic therapy	15	18	17	ns	15	17	ns
Modifiable Risk factors							
*High SBP**	68	62	54	ns	68	60	ns
High LDL-C	49	47	49	ns	50	50	ns
Smoking	20	19	23	ns	20	12	ns
*High BMI**	19	19	23	ns	19	20	ns
*High FPG**	56	54	56	ns	56	54	ns
*Low HDL-C**	29	29	22	ns	29	27	ns
*High TG**	25	27	19	ns	25	25	ns
Metabolic Syndrome	32	28	27	ns	33	28	ns
Laboratory clinical-chemistry data							
Total-C, mg/dL	192±47	187±49	187±49	ns	192±47	187±49	ns
HDL-C, mg/dL	54±18	51±16	51±15	ns	54±18	51±16	0.04
LDL-C, mg/dL	115±38	112±39	112±48	ns	115±38	111±39	ns
TG, mg/dL	115±64	122±65	116±59	ns	115±64	121±64	ns
ApoA1, mg/dL	137±41	125±42	131±36	ns	137±41	126±41	0.04
TG/HDL-C	2.5±.1.9	2.7±1.8	2.5±1.6	0.05	2.5±1.9	2.6±1.7	0.02
Glucose, mg/dL	106.1±27.9	106±31	101±19	ns	106.1±27.9	105±28	ns
Insulin, µUI/mL	10.2±108	9.89.2	8.3±5.3	ns	10.2±10.8	9.5±8.6	ns
HOMA index	2.9±4.3	2.8±2.8	2.1±1.5	ns	2.9±4.3	2.6±2.6	ns

Continuous variables are presented as mean±standard deviation, categorical variables as (%)

CAD: coronary artery disease; high SBP (SBP=systolic blood pressure): SBP ≥130/85 mmHg or antihypertensive medication; Total-C (C=cholesterol); high LDL-C (LDL-C=LDL cholesterol): LDL-C>115mg/dL;

high BMI (BMI= body mass index): BMI > 30 Kg/m2; high FPG (FPG=fasting plasma glucose): high FPG: diabetes or FPG > 100 mg/dL; low HDL-C (HDL-C=HDL cholesterol): HDL-C <50 mg/dl in women and <40 mg/dl in men or specific treatment for this lipid abnormality; high TG (TG= triglycerides): TG ≥150 mg/dl or specific treatment for this lipid abnormality; ApoA1= Apolipoprotein 1.

*= component of metabolic syndrome

Table 2 Suppl mat. *Predictors of obstructive CAD and inducible myocardial ischemia at multivariate analyses by logistic regression analysis*

	**Obstructive CAD**	**Inducible Myocardial Ischemia**
	Multivariate analysis OR (CI 95%), p-value	***Multivariate analysis OR (CI 95%), p-value***
**Age**	1.1(1.0-1.11), p<0.0001	1.0(1.0-1.1), p=0.01
**Male sex**	4.7(2.9-7.6), p<0.0001	1.9(1.1-3.1), p=0.01
**Family History of CAD**	ns	0.6)0.4-1.1), p=0.07
**Metabolic Syndrome**	1.4(0.9-2.2), p=0.1	0.7(0.4-1.2), p=0.2
**High LDL-C**	ns	0.6)0.4-1.1), p=0.07
**Smoking**	1.8(1.1-3.1), p=0.03	1.4(0.8-2.5), p=0.3
**eNOS Asp genotype**	-	2.1(1.3-3.4), p=0.003

ns= not significant

CAD: coronary artery disease; high LDL-C (LDL-C=LDL cholesterol): LDL-C >115mg/dL

Table 3 Suppl mat: *Comparison of clinical characteristics, modifiable risk factors and laboratory clinical-chemistry data among patient groups defined by absence/presence of obstructive CAD and absence/presence of inducible MI*

	no CAD- no MI n=87	CAD and MI n=84	p-value
Clinical characteristics			
Age, years	59.1±9.3	63.6±8.9	0.001
Male gender	49	84	<0.0001
Family history of CAD	53	49	ns
LVEF (%)	62±5	60±5	0.006
Symptoms			
Typical Angina	40	56	0.04
Medications			
Antihypertensives and Anti-ischemics	70	63	ns
Aspirin	52	48	ns
Statins	29	29	ns
Anti diabetic therapy	21	17	ns
Modifiable Risk factors			
High SBP	29	34	ns
High LDL-C	61	39	0.003
Smoking	22	25	ns
High BMI	31	12	0.002
High FPG	53	52	ns
Low HDL-C	10	39	<0.0001
High TG	22	36	0.04
Metabolic Syndrome	29	34	ns
Laboratory clinical-chemistry data			
Total-C, mg/dL	212.7±41.5	177.8±49.1	<0.0001
LDL-C, mg/dL	129.1±34.1	109.0±40.7	0.0002
HDL-C, mg/dL	61.0±18.2	44.4±12.7	<0.0001
TG, mg/dL	114.9±54.2	130.0±6401	ns
ApoA1, mg/dL	138.8±43.5	120.4±37.3	0.01
TG/HDL	2.1±1.3	3.1±1.9	<0.0001
Glucose, mg/dL	108.2±33.2	104.1±34.5	ns
Insulin, µUI/mL	8.9±5.3	9.2±8.4	ns
HOMA Index	2.5±1.9	2.5±2.5	ns
Genotype distribution			

Glu/Glu	58	42	
Glu/Asp	33	45	ns
Asp/Asp	9	13	
Glu/Glu	57	42	0.03
Asp	43	58	

Continuous variables are presented as mean±standard deviation, categorical variables as (%)

CAD: coronary artery disease; MI: myocardial ischemia ; high SBP (SBP=systolic blood pressure): SBP ≥130/85 mmHg or antihypertensive medication; Total-C (C=cholesterol); high LDL-C (LDL-C=LDL cholesterol): LDL-C >115mg/dL; high BMI (BMI= body mass index): BMI > 30 Kg/m2; high FPG (FPG=fasting plasma glucose): high FPG: diabetes or FPG > 100 mg/dL; low HDL-C (HDL-C=HDL cholesterol): HDL-C <50 mg/dl in women and <40 mg/dl in men or specific treatment for this lipid abnormality; high TG (TG= triglycerides): TG ≥150 mg/dl or specific treatment for this lipid abnormality; ApoA1= Apolipoprotein 1.

*= component of metabolic syndrome
